# Phosphorylation and Alternative Translation on Wheat Germ Cell-Free Protein Synthesis of the DHBV Large Envelope Protein

**DOI:** 10.3389/fmolb.2019.00138

**Published:** 2019-12-03

**Authors:** Guillaume David, Marie-Laure Fogeron, Roland Montserret, Lauriane Lecoq, Adeline Page, Frédéric Delolme, Michael Nassal, Anja Böckmann

**Affiliations:** ^1^Institut de Biologie et Chimie des Protéines, MMSB, Labex Ecofect, UMR 5086 CNRS, Université de Lyon, Lyon, France; ^2^Protein Science Facility, SFR BioSciences CNRS UMS3444, Inserm US8, UCBL, ENS de Lyon, Lyon, France; ^3^Internal Medicine II/Molecular Biology, University Hospital Freiburg, Freiburg, Germany

**Keywords:** cell-free protein synthesis (CFPS), wheat-germ, phosporylation, alternative translation initiation, HBsAg—surface antigen of hepatitis B virus

## Abstract

Wheat-germ cell-free protein synthesis (WG-CFPS) is a potent platform for the high-yield production of proteins. It is especially of interest for difficult-to-express eukaryotic proteins, such as toxic and transmembrane proteins, and presents an important tool in high-throughput protein screening. Until recently, an assumed drawback of WG-CFPS was a reduced capacity for post-translational modifications. Meanwhile, phosphorylation has been observed in WG-CFPS; yet, authenticity of the respective phosphorylation sites remained unclear. Here we show that a viral membrane protein, the duck hepatitis B virus (DHBV) large envelope protein (DHBs L), produced by WG-CFPS, is phosphorylated upon translation at the same sites as DHBs L produced during DHBV infection of primary hepatocytes. Furthermore, we show that alternative translation initiation of the L protein, previously identified in virus-producing hepatic cells, occurs on WG-CFPS as well. Together, these findings further strengthen the high potential of WG-CFPS to include the reproduction of specific modifications proteins experience *in vivo*.

## Introduction

Wheat germ cell-free protein synthesis (WG-CFPS) is an alternative method to cell-based protein expression. Exploiting the high translation efficiency of the plant ribosome, protein yields are often higher than in most other eukaryotic cell-free extracts, such as rabbit reticulocyte lysate (RRL) (Schweet et al., 1958), human-derived cells (Weber et al., 1975), or insect cells (Smith et al., 1983) (for a recent review, see Zemella et al., 2015). However, reduced or even lacking post-translational modifications have long been considered a drawback of WG-CFPS compared to other systems, as small cofactors required for the activity of certain enzymes might be eliminated during extract preparation. Nonetheless, most kinases do not need cofactors, and a proteomics study on wheat germs from *Triticum aestivum* has highlighted the presence of at least 12 different kinases which do not require cofactors for activity, including several serine/threonine kinases (Mak et al., 2006). Thus, even though the exact enzyme content of cell-free extracts from wheat germ remains to be determined, altogether these data suggest that phosphorylation can in principle occur. Indeed, background phosphorylation in WG-CFPS has been mentioned since (Harbers, 2014), and first solid evidence for extensive phosphorylation on WG-CFPS has been provided recently for the hepatitis C virus NS5A protein (Badillo et al., 2017). Still, it remained unclear if the observed phosphorylation patterns were identical to those produced in authentic replication models, also because the precise authentic NS5A phosphorylation sites remain enigmatic (Badillo et al., 2017).

We here investigated the large envelope protein (DHBs L) from duck hepatitis B virus (DHBV). DHBV has been extensively studied as a model for the human HBV (Schultz et al., 2004), and remains a valuable subject in recent evolutionary and therapeutic studies (Noordeen et al., 2015; Zheng et al., 2017). The two viruses bear some differences, notably concerning the envelope proteins that occur in three forms in HBV, but only in two in DHBV: the small DHBs S and the large DHBs L proteins, that differ from each other by the addition of the DpreS domain at the N-terminal of the S protein. DHBs S is predicted to possess 3 or 4 transmembrane domains (Stirk et al., 1992; Schultz et al., 2004), and shares 30% sequence homology with the human virus small envelope protein HBs S. DpreS is involved in a variety of interactions in the viral life cycle, and its counterpart in the human virus has been described as predominantly natively unstructured (Chi et al., 2007; Jürgens et al., 2013). In both HBV and DHBV, the N-terminal part of the L protein is myristoylated *in vivo* to allow for preS anchorage to the membrane (Macrae et al., 1991).

DHBs L has been described to be phosphorylated: on SDS-PAGE and Western Blots (WBs), the protein generally appears at an approximate size of 35.5 kDa, with a second band migrating around 36 kDa, and in some cases even a third band appearing at 37 kDa (Grgacic and Anderson, 1994). These three forms, which have been called, respectively, p35, p36, and p37, have been shown to originate from different phosphorylation states of the protein by *in cellulo* studies using site-directed mutagenesis (Grgacic et al., 1998). These studies defined the main phosphorylation sites, showing that 64% of all incorporated phosphate groups were located on S118, which would correspond to the p36 form, and 17% on T79, T89, S117, and T155, which together with phosphorylation on S118 would induce the p37 form. The investigated sites all exhibit the minimal consensus target sequence for mitogen-activated-protein (MAP) kinases, Ser/Thr-Pro, with S118 even showing the optimal sequence Pro-X-Ser/Thr-Pro (Davis, 1993), explaining the prevalence of this site for phosphorylation. DHBs L protein phosphorylation is not required for infectivity (Grgacic et al., 1998), but phosphorylation of S118 possibly plays a role in modulation of viral replication *via* gene transactivation of the host cell (Rothmann et al., 1998).

Additional lower molecular weight bands have been identified previously for the DHBs envelope proteins (Schlicht et al., 1987; Fernholz et al., 1993; Grgacic and Anderson, 1994) which could not be accounted for by post-translational modifications. These minor proteins of 35, 33, and 30 kDa have been ascribed to alternative translation products, starting from internal initiation codons (respectively M9, M28, and M53) and thus lacking the respective upstream parts of DHBs L. Another major form of ~28 kDa was shown to result from proteolysis rather than alternative initiation (Fernholz et al., 1993).

We here show that DHBs L synthesized via WG-CFPS is phosphorylated on the same sites as previously pinpointed by mutagenesis in animal cells (Grgacic et al., 1998) as well as on two additional sites which have not been identified before, as previous work focused only on MAP kinase consensus sequences. In addition, protein forms from alternative translation initiation were also detected upon WG-CFPS. Both observations highlight the high potential of this system to produce proteins with similar modifications as those observed in complex cellular systems.

## Materials and Methods

### Plasmids

cDNA of DHBs L (DHBV Strain United States; UniProt accession number P03145/ENA X12798) was amplified by PCR and cloned into pEU-E01-MCS vector (CellFree Sciences, Japan). The resulting plasmid was transformed into *E. coli* competent cells (TOP10, Life Technologies). DNA was made using a NucleoBond Xtra Maxi kit (Macherey-Nagel, France). Plasmids were purified using phenol/chloroform extraction, as described by CellFree Sciences (Yokohama, Japan).

### Wheat Germ Cell-Free Protein Synthesis

Wheat germ extract were home-made and prepared using untreated durum wheat seeds (Semences du Sud, France) (Takai et al., 2010; Fogeron et al., 2017). Cell-free synthesis was performed with uncoupled transcription and translation. Transcription was performed using 100 ng/μl plasmid, 2.5 mM NTP mix (Promega, France), 1U/μl RNase inhibitor (CellFreeSciences, Japan) and 1U/μl SP6 RNA polymerase (CellFreeSciences, Japan) in a buffer containing 80 mM Hepes-KOH pH 7.6, 16 mM magnesium acetate, 10 mM DTT and 2 mM spermidine (CellFree Sciences, Japan). The solution was incubated for 6–7 h at 37°C, then the produced mRNA was used for translation. For the small-scale expression test, translation was done in 96-well plates while for the affinity purification, translation was performed in 6-well plates, in order to obtain higher amounts of protein. In both cases, translation was done using the so-called bilayer method (Takai et al., 2010; Fogeron et al., 2015a; David et al., 2018). The feeding buffer composition was 30 mM HEPES-KOH pH 7.8, 4 mM DTT, 0.25 mM GTP, 1.2 mM ATP, 0.4 mM spermidine, 16 mM creatine phosphate, 2.7 mM magnesium acetate, 100 mM potassium acetate, 25 mM amino acid mix, and 0.1% (w/v) Maltyl-Neopentyl Glycol (MNG-3). The translation mix, containing mRNA, wheat germ extract (20 μl for the 96-well plate reaction, 250 μl for the 6-well plate reaction), 6 mM amino acid mix, 4 μg/ml creatine kinase and the corresponding detergent was then deposited under the feeding buffer at the bottom of the well, allowing for the formation of a bilayer, the WGE having a higher density than the buffer. The plate was incubated for 16 h at 22°C without shaking.

### Affinity Chromatography Purification

The resulting solution after synthesis in 1-well of a 6-well plate in presence of 0.1% MNG-3 was incubated with 2 U/μl benzonase for 30 min on a rolling wheel at RT. Afterwards, the total fraction from the cell-free synthesis was centrifuged at 20 000 *g*, 4°C for 30 min. The supernatant was loaded on 1-mL *Strep*-Tactin^©^ Superflow^©^ gravity flow columns (IBA Lifesciences, Germany). Purification was done as described in the manual, with all buffers containing 0.1% (w/v) DDM, as described previously (Fogeron et al., 2015b, 2016). The protein of interest was eluted in 100 mM Tris-HCl pH 8.0, 150 mM NaCl, 1 mM EDTA, 2.5 mM D-desthiobiotin and 0.1% DDM.

### SDS-PAGE and Western Blotting Analysis

Experiments were evaluated using 15% polyacrylamide SDS-PAGE gels as described in Fogeron et al. (2015a). Samples were resuspended in a loading buffer containing 62.5 mM Tris-HCl pH 6.8, 10% glycerol (v/v), 2% SDS (w/v), 5% β-mercaptoethanol (v/v) and 0.01% bromophenol blue (w/v), and incubated for 15 min before loading. Western blotting of DHBs L was carried out by protein transfer onto a nitrocellulose membrane through iBlot2^©^ gel transfer. The nitrocellulose membrane was then blocked with 5% non-fat milk powder in PBS-T buffer, which contains 12 mM sodium phosphate pH 7.4, 137 mM NaCl, 2.7 mM KCl, 0.05% Tween^©^ 20 (v/v). The membrane was then incubated with a rabbit anti-DHBs primary antibody for 1 h and incubated with an anti-rabbit IgG HRP conjugated secondary antibody (Promega, France) for 1 h. Epitope-containing bands were observed using a Amersham ECL Prime Western Blotting Detection Reagent kit (GE Healthcare, France). All incubations were carried out at room temperature.

### In-gel Digestion for Mass Spectrometry (MS)

Protein sample in gel was excised from SDS-PAGE and in-gel reduction, alkylation and digestion was applied. The gel band was reduced with 10 mM DTT in 100 mM NH_4_HCO_3_ (Sigma Aldrich) for 1 h at 57°C and alkylated for 1 h in the dark with 55 mM iodoacetamide in 100 mM NH_4_HCO_3_ (Sigma Aldrich), washed in 25 mM NH_4_HCO_3_, dehydrated with acetonitrile and dried in a speed-vac. Then the gel pieces were rehydrated with 20 μl trypsin solution 12.5 ng/μl in 50 mM NH_4_HCO_3_ (trypsin porcine, PROMEGA) for 1 h on ice and incubated in 50 mM NH_4_HCO_3_ overnight at 37°C. The peptides were extracted twice with 50 μl of acetonitrile/water/formic acid-45/45/10 (v/v/v) followed by a final extraction with 50 μl of acetonitrile/formic acid (FA)-95/05 (v/v). Peptides were dried in a speed-vac before nanoLC-MS/MS analysis and then suspended in 10 μl 0.1% HCOOH.

### NanoLC-MS/MS Analysis

Samples analysis used an Ultimate 3000 nano-RSLC (Thermo Scientific, San Jose California) coupled (on line) with a Q Exactive HF mass spectrometer by a nano-electrospray ionization source (Thermo Scientific, San Jose California). Three microliter of peptide mixtures were added on a C18 Acclaim PepMap100 trap-column (75 μm ID × 2 cm, 3 μm, 100 Å, Thermo Fisher Scientific) with 2% ACN, 0.05% TFA in H_2_O at 5 μl/min for 3.0 min and separated on a C18 Acclaim Pepmap100 nano-column, 50 cm × 75 μm i.d, 2 μm, 100 Å (Thermo Scientific). A linear 60 min gradient (3.2% to 40% buffer B) (A: 0.1% FA in H_2_O, B: 0.1% FA in ACN) followed by a 2 min gradient (40–76% of B) was used, was hold for 10 min and then returned to the initial conditions within 1 min, for 14 min. This was done for a total duration of 90 min, at a flow rate of 300 nl/min. The oven temperature was 40°C.

The results were analyzed with the TOP20 HCD method. The scans were acquired in a data dependent strategy selecting the fragmentation events, that were based on the 20 most abundant precursor ions in the survey scan (375–1,600 Th). The survey scan had a resolution of 60,000 at m/z 200 Th. Survey scans had Ion Target Values of 3E6 and 1E5, respectively, for the Orbitrap for the MS^2^ mode. The maximum injection time was to 60 ms for both. Acquisition parameters for HCD MS/MS spectra were the following: collision energy, 27; isolation width, 2 m/z. Precursors with unknown charge state, or a unit charge state were excluded. Peptides selected for MS/MS acquisition were then put on an exclusion list (for 20 s) via the dynamic exclusion mode in order to limit spectra duplication.

### Mass Spectrometry Data Analysis

Proteins identification was done using database searching through Sequest HT (Eng et al., 1994) and MS Amanda (Dorfer et al., 2014), and using the Proteome Discoverer 2.2 software (Thermo Scientific) against the Swissprot DHBV sequence. The precursor mass tolerance was 10 ppm, and fragment mass tolerance was 0.02 Da. Up to 2 missed cleavages were allowed. Variable modification were oxidation (M), acetylation (Protein N-terminus) and Phosphorylation (S, T, Y). Carbamidomethylation (C) was set as fixed modification. Peptides were filtered with a fixed value PSM validator and rank 1. Manual validation of phosphorylation sites was then done.

## Results and Discussion

### Alternative Translation Initiation Is Observed on DHBs L WG-CFPS

**Figure 1:**
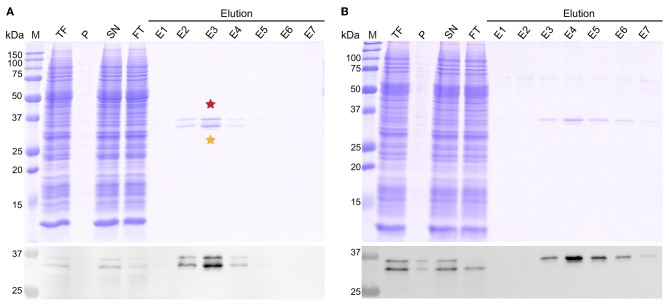
StrepTactin^©^ affinity purification of DHBs L with a StrepTag on **(A)** the C-terminal end or on **(B)** the N-terminal end. Coomassie blue gel and western blot using an anti-DHBs S antibody are shown. Purification was performed in the presence of 0.1% DDM. M, molecular weight marker; TF, total fraction; P, pellet; SN, supernatant; FT, column flow-through; E1-E7, elution fractions. Comparable volumes were loaded on the gel for all fractions. Bands marked with a star were cut out from the gel for mass spectrometry analysis.

We have recently reported WG-CFPS of DHBs S, which autoassembles during expression in structures called subviral particles (SVPs) (David et al., 2018). SVPs are produced in large amounts *in vivo*, and are there constituted by about 70% DHBs S and 30% DHBs L (m/m) (Schultz et al., 2004). Because expression of DHBs L alone does not lead to formation of SVPs, we here aimed at expressing it in a detergent-solubilized form. WG-CFPS of DHBs L was thus carried out using the bilayer method (Takai et al., 2010) in the presence of 0.1% (w/v) MNG-3 (Chae et al., 2010; Fogeron et al., 2015a). Two constructs of DHBs L were used, carrying either a StrepTag II (Schmidt and Skerra, 2007) on the N-terminal or on the C-terminal end of the protein. We produced the protein in 1-well of a 6-well plate reaction, yielding 0.11 mg of protein, which corresponds to 0.44 mg DHBs L per ml of WGE. The presence of the detergent MNG-3 allowed to produce DHBs L in a solubilized form, as presented in Figure 1 where the SDS-PAGE and western blots of the total, pellet, and supernatant fractions are shown for both constructs. The elution fractions from affinity purification of DHBs L using the StrepTag on the C-terminal or N-terminal end are shown as well in Figure 1. It can be seen that for DHBs L with the StrepTag at the C-terminal end, two separate bands are observed for all DHBs L-containing fractions, which show comparable intensities. Interestingly, for the construct with the N-terminal StrepTag (Figure 1B), under the same conditions, only the upper band could be purified, while the lower band was located in the flow through fraction. This suggests that the lower band results from alternative translation initiation, which has previously been described to result in two additional DHBs L bands, p33 and p30 (Fernholz et al., 1993). This hypothesis is supported by the lack of recognition of the lower band by an antibody targeting a linear epitope within amino acids 2–26 (Figure S1). Alternative initiation could start at M9, M28, or M53, yielding proteins that are, respectively 837, 2,960, or 5,764 Da smaller than the full-length protein. Leaky scanning of ribosomes could be at the origin of this alternative translation mechanism, as such a model has already been proposed to explain production of the three envelope proteins (Zajakina et al., 2004) and the polymerase (Fouillot et al., 1993; Chen et al., 2014) of HBV. Another possibility is ribosomal shunting (Fütterer et al., 1993), during which ribosomes bind to the 5′ end of the messenger RNA and skip during the scan parts of the message, a mechanism proposed for translation of different viral proteins, including the DHBV polymerase (Sen et al., 2004). Proteolytic degradation is not formally excluded but rather implausible considering that no other degradation products are detected and that the main processing product described in the literature produces a band of ± 28 kDa (Fernholz et al., 1993), which is not observed here.

### Phosphorylation of Specific Sites Is Observed on DHBs L WG-CFPS

**Figure 2:**
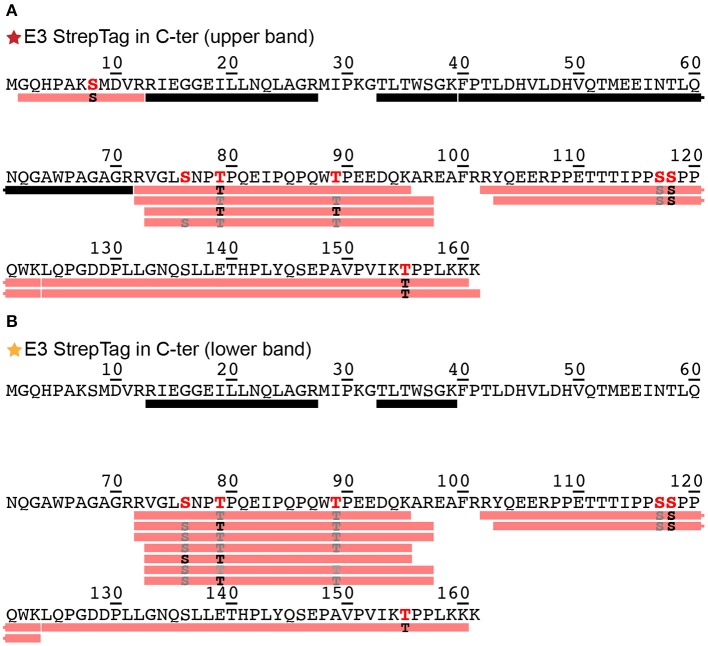
Identification by mass spectrometry of phosphorylation sites shown on the DpreS domains from DHBs L. Analyses have been performed on the upper **(A)** and lower **(B)** bands of the E3 fraction resulting from the purification of DHBs L (Figure 1A). Black bars represent analyzed peptides devoid of phosphorylation as identified by mass spectrometry. The detailed information about the identified peptides bearing a phosphorylation are available in Figure S2. Pink bars represent peptides where one or more phosphorylation sites have been confirmed; the residues in black correspond to the phosphorylation sites formally identified (in bold red in the sequence), while the ones in gray correspond to possible phosphorylation sites with a lower degree of confidence (in red in the sequence). MS/MS spectra for each identified phosphorylation sites of the upper band **(A)** can be found in Figure S2.

Phosphorylation of DHBs L protein in primary duck hepatocyte cultures has been shown using western blotting and radiolabeling (Grgacic and Anderson, 1994). Since migration on gels as multiple bands can as well be an indication of different phosphorylation patterns (Ubersax et al., 2003), we evaluated this possibility by subjecting the two main bands of the E3 fraction (Figure 1A, red and orange stars) to analysis by mass spectrometry. The bands were cut from the gel, reduced, alkylated, digested and analyzed using a nanoRSLC-Q Orbitrap (Q Exactive HF, Thermo Scientific) mass spectrometer. We assessed phosphorylation using the PD2.2 software in association with the two search engines MS Amanda and Sequest HT. The analysis revealed the presence of DHBs L (P03145, strain United States) in both bands, and showed a total sequence coverage of 48% for the upper band and 36% for the lower one, with peptide fragments stemming mainly from the DpreS part of the protein (amino acids 1–161) and only a very small part from the C-terminal S domain (amino acids 185–191). The reason of the poor sequence coverage in the S domain likely lies in its hydrophobic nature and resulting protection by detergent molecules. For the upper band (Figure 2A, only the sequence of DpreS is shown), the MS data validated 6 phosphorylation sites: S8, S76, T79, T89, S118, T155 (for individual spectra, see Figure S2). A possible additional site is proposed on S117. For the lower band (Figure 2B), phosphorylation sites are identified as well and validated for 5 residues (S76, T79, T89, S118, T155), with an additional possibility on S117. These results show that both bands actually correspond to phosphorylated DHBs L. Moreover, while the coverage starts at residue G2 for the upper band, it only starts at R13 for the lower band. This observation supports the alternative translation from M9 as origin of the lower band (Fernholz et al., 1993), which would match with the difference of 837 Da observed on the SDS-PAGE.

The phosphorylation sites observed are consistent with those identified previously (Grgacic et al., 1998), which highlighted S118 as major (64 %), and T79, T89, S117, and T155 as minor (17%) phosphorylation sites, accounting for 81% of total phosphorylation altogether.

While mass spectrometry experiments do not allow to directly quantify, the number of peptide-spectrum matches (PSM) shown in Figure S2G for the different peptides corresponding to the different phosphorylations gives a rough estimate. From a total of 53 PSMs, 35 are observed for S118, 9 in total for T79, T89, and T155, and 9 for S8 and S76. This corresponds to 66, 17, and 17% of total PSM, which matches quite well with the percentages determined in cells. The newly identified phosphorylation sites S8 and S76 might therefore account for the 19% unassigned phosphorylation events from this previous study (Grgacic et al., 1998). Indeed, analysis of these sites was omitted in the study by Grgacic et al., since these residues do not show the consensus sequence for MAP kinases (Ser/Thr-P), and the authors therefore did not test constructs showing these point mutations to investigate resulting phosphorylation patterns. All previously investigated sites were shown as nonessential for infectivity; however, a potential role for S8 and S76 phosphorylation remains to be investigated, as these residues were not addressed before.

## Conclusion

While protein phosphorylation as such in the wheat-germ cell-free system has recently been described (Harbers, 2014; Badillo et al., 2017), we here showed that the phosphorylation sites, obtained on WG-CFPS of the DHBV HBs L envelope protein, match the same five sites pinpointed in a prior *in cellulo* analysis (Grgacic et al., 1998) that focused on MAP kinases target sites. In addition, we identified two new previously unnoted sites. The observed phosphorylation of the DHBV HBs L protein confirms that active kinases, likely from the MAP family, are present in the wheat germ extract. We moreover showed that alternative translation initiation, enabling eukaryotic cells to access different isoforms using the same mRNA (Kochetov et al., 2005; Bazykin and Kochetov, 2011; Hopkins et al., 2013), is not restricted to rabbit reticulocyte lysate, as previously described (Liang et al., 1996), but also takes place in the wheat-germ cell-free system. Our work adds new potential to the already diverse panel of possibilities given by protein synthesis in the wheat-germ cell-free system, and shall broaden its applications in the context not only of functional, but also structural studies of proteins carrying modifications.

## Data Availability Statement

All datasets generated for this study are included in the article/Supplementary Material.

## Author Contributions

GD and M-LF implemented and conducted cell-free protein synthesis. AP and FD did mass spectrometry analyses and designed the corresponding figures. GD, M-LF, RM, LL, AP, and FD analyzed the data. MN contributed reagents and expertise in DHBV. M-LF and AB designed and supervised the study. GD, M-LF, and AB wrote the manuscript. All authors contributed to the manuscript and approved the submitted version.

### Conflict of Interest

The authors declare that the research was conducted in the absence of any commercial or financial relationships that could be construed as a potential conflict of interest.
