# Supplementary material for: Phosphorylation and Alternative Translation on Wheat Germ Cell-Free Protein Synthesis of the DHBV Large Envelope Protein
Source: Front Mol Biosci. 2019 Dec 3;6:138. doi: 10.3389/fmolb.2019.00138 (PMC6902406; doi:10.3389/fmolb.2019.00138)
Supplement: Supplementary file 1 [file Data_Sheet_1.PDF]

SUPPLEMENTARY MATERIAL

Phosphorylation and alternative translation on wheat germ cell-free protein synthesis of the DHBV large envelope protein

Guillaume David[1], Marie-Laure Fogeron[1], Roland Montserret[1], Lauriane Lecoq[1], Adeline Page[2], Frédéric Delolme[2], Michael Nassal[3], & Anja Böckmann[1*]

[1]Institut de Biologie et Chimie des Protéines, MMSB, Labex Ecofect, UMR 5086 CNRS, Université de Lyon, 7 passage du Vercors, 69367 Lyon, France

[2]Protein Science Facility, SFR BioSciences CNRS UMS3444, Inserm US8, UCBL, ENS de Lyon, 50 Avenue Tony Garnier, 69007 Lyon, France

[3]University Hospital Freiburg, Internal Medicine II / Molecular Biology, Hugstetter Str. 55, D-79106 Freiburg, Germany

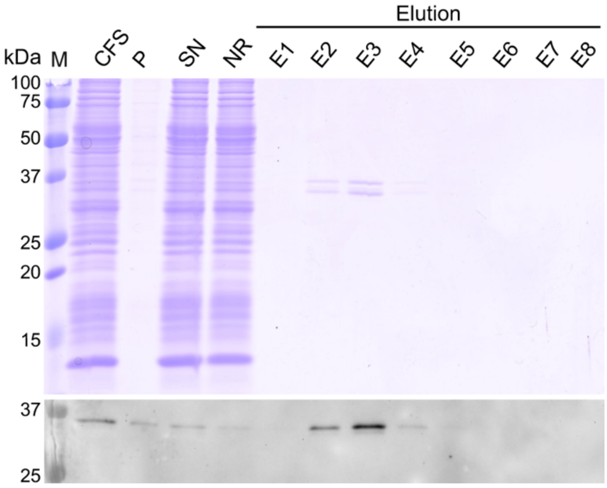

Fig. S1. The lower band is not recognized by an antibody targeting at a linear epitope within DHBs L protein amino acids 2-26. StrepTactin© affinity purification of DHBs L with a StrepTag on the C-terminal end. Coomassie blue gel and western blot using an anti-DpreS aa 2-26 antibody are shown. Purification has been performed in the presence of 0.1% DDM. M, molecular weight marker; TF, total fraction; P, pellet; SN, supernatant; FT, column flow-through; E1-E7, elution fractions. Comparable volumes were loaded on the gel for all fractions.

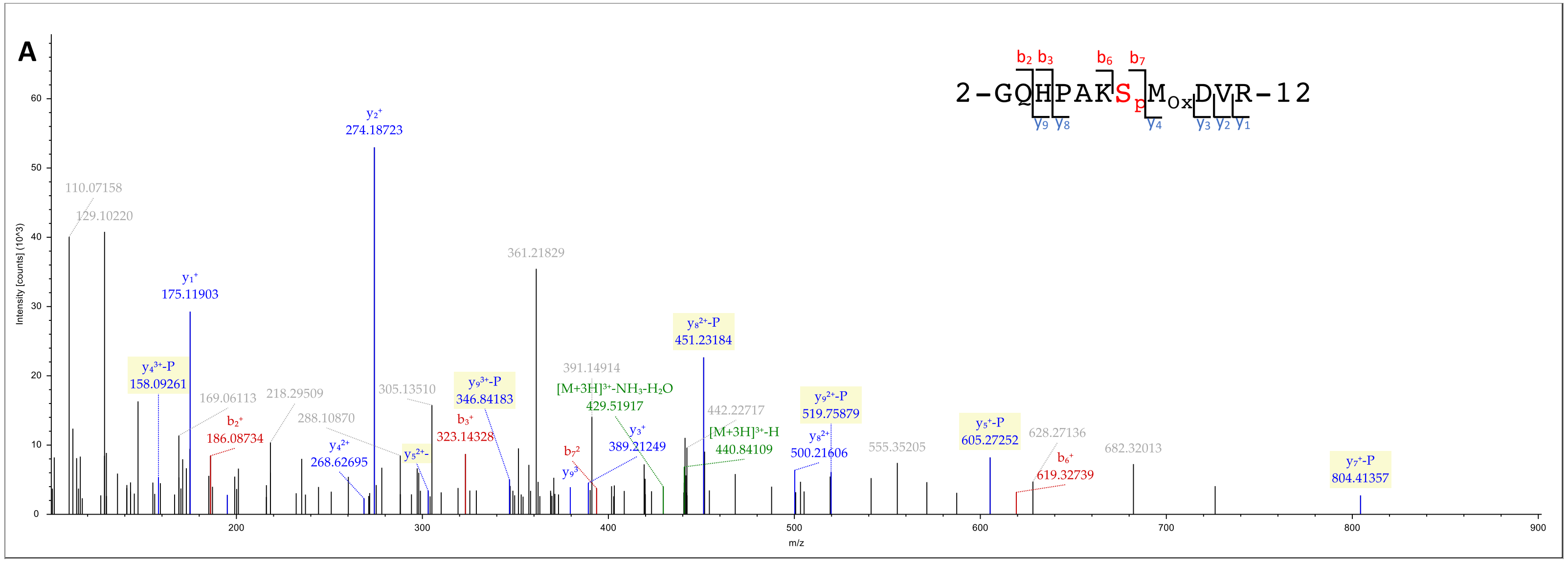

A
2-GQHPAKS$_p$M$_{Ox}$DVR-12
Intensity [counts] (10^3)
m/z
110.07158
129.10220
y_4^{3+}-P 158.09261
169.06113
y_1^+ 175.11903
b_2^+ 186.08734
218.29509
y_4^{2+} 268.62695
y_2^+ 274.18723
288.10870
y_5^{2+}-
305.13510
b_3^+ 323.14328
y_9^{3+}-P 346.84183
361.21829
b_7^2
y_9^3
y_3^+ 389.21249
391.14914
$[M+3H]^{3+}$-NH_3-H_2O 429.51917
$[M+3H]^{3+}$-H 440.84109
442.22717
y_8^{2+}-P 451.23184
y_8^{2+} 500.21606
y_9^{2+}-P 519.75879
555.35205
y_5^+-P 605.27252
b_6^+ 619.32739
628.27136
682.32013
y_7^+-P 804.41357

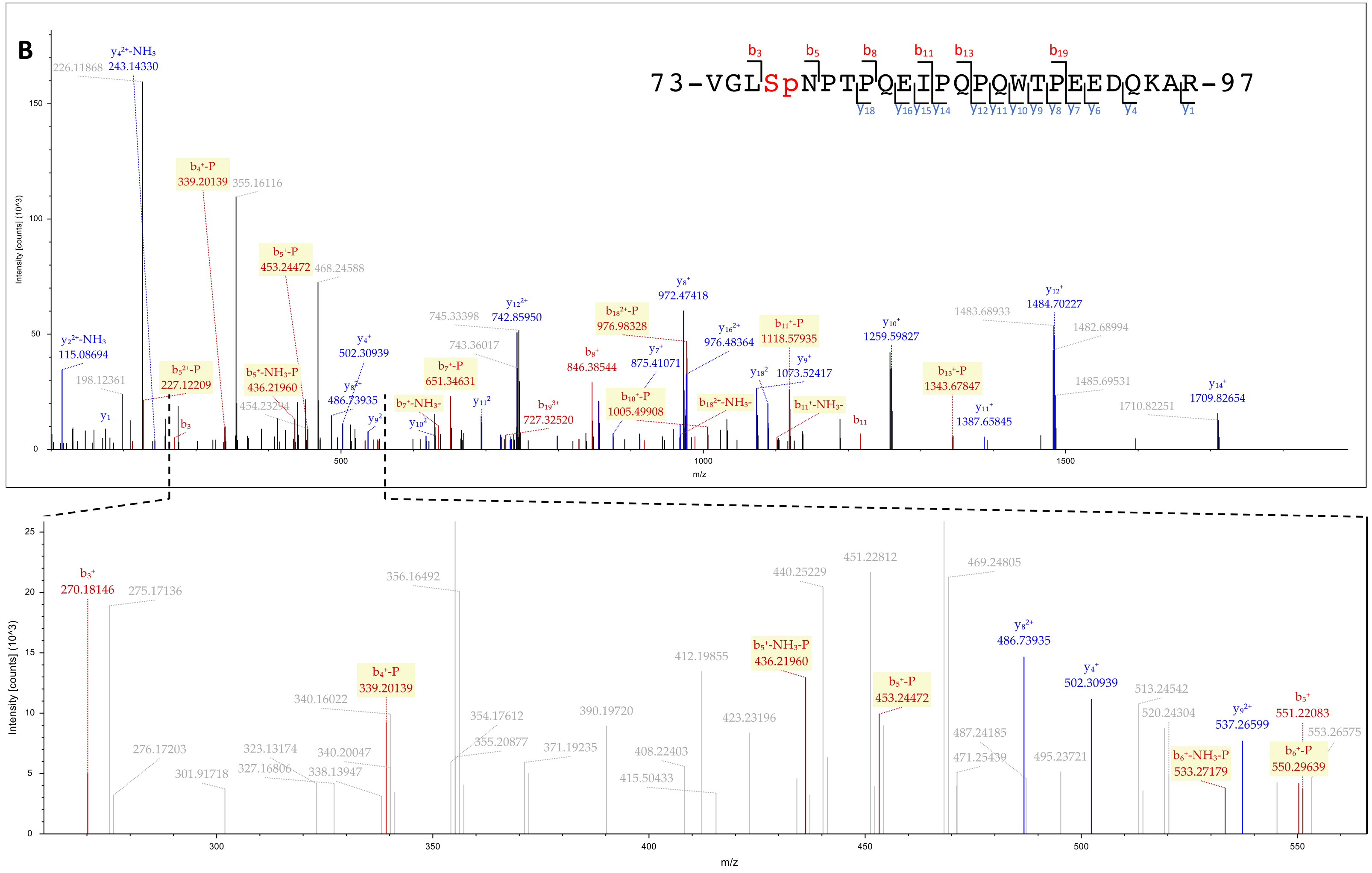
B
73-VGLSpNPTPQEIPQPQWTPEEDQKAR-97
b3 b5 b8 b11 b13 b19
y18 y16 y15 y14 y12 y11 y10 y9 y8 y7 y6 y4 y1
Intensity [counts] (10^3)
m/z
y4²⁺-NH3 243.14330
226.11868
y2²⁺-NH3 115.08694
198.12361
b5²⁺-P 227.12209
b4⁺-P 339.20139
355.16116
b5⁺-P 453.24472
468.24588
b5⁺-NH3-P 436.21960
454.23294
y4⁺ 502.30939
y8²⁺ 486.73935
b7⁺-P 651.34631
b7⁺-NH3-
745.33398
743.36017
y12²⁺ 742.85950
b19³⁺ 727.32520
b8⁺ 846.38544
y7⁺ 875.41071
b10⁺-P 1005.49908
b18²⁺-P 976.98328
y8⁺ 972.47418
y16²⁺ 976.48364
b18²⁺-NH3-
b11⁺-P 1118.57935
y9⁺ 1073.52417
b11⁺-NH3-
b11
y10⁺ 1259.59827
b13⁺-P 1343.67847
y11⁺ 1387.65845
1483.68933
y12⁺ 1484.70227
1482.68994
1485.69531
1710.82251
y14⁺ 1709.82654
b3⁺ 270.18146
275.17136
276.17203
301.91718
323.13174
327.16806
340.20047
338.13947
340.16022
356.16492
354.17612
355.20877
371.19235
390.19720
408.22403
415.50433
412.19855
423.23196
b5⁺-NH3-P 436.21960
440.25229
451.22812
b5⁺-P 453.24472
469.24805
487.24185
471.25439
495.23721
y8²⁺ 486.73935
y4⁺ 502.30939
513.24542
520.24304
b6⁺-NH3-P 533.27179
y9²⁺ 537.26599
b6⁺-P 550.29639
b5⁺ 551.22083
553.26575

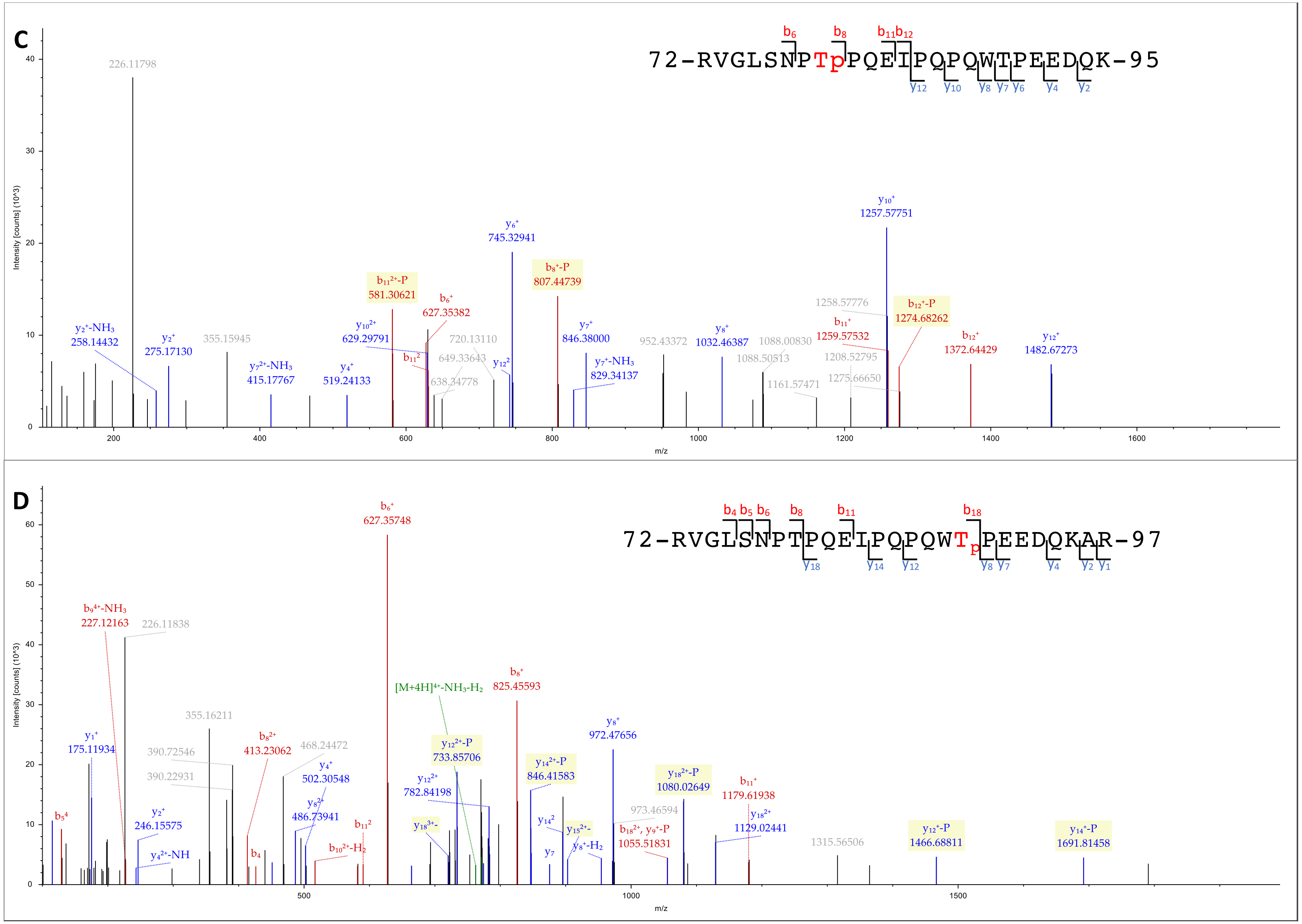
C
72-RVGLSNPTpPQEIPQPQWTPEEDQK-95
Intensity [counts] (10^3)
m/z
226.11798
y10+ 1257.57751
y6+ 745.32941
b11 2+-P 581.30621
b8+-P 807.44739
b6+ 627.35382
b12+-P 1274.68262
y2+-NH3 258.14432
y2+ 275.17130
355.15945
y10 2+ 629.29791
720.13110
y7+ 846.38000
952.43372
y8+ 1032.46387
1088.00830
1258.57776
b11+ 1259.57532
b12+ 1372.64429
y12+ 1482.67273
y7 2+-NH3 415.17767
y4+ 519.24133
649.33643
y7+-NH3 829.34137
1088.50513
1208.52795
638.34778
1161.57471
1275.66650
D
72-RVGLSNPTPQEIPQPQWTpPEEDQKAR-97
b6+ 627.35748
b9 4+-NH3 227.12163
226.11838
b8+ 825.45593
[M+4H]4+-NH3-H2
355.16211
y1+ 175.11934
390.72546
390.22931
b8 2+ 413.23062
468.24472
y4+ 502.30548
y8 2+ 486.73941
y12 2+-P 733.85706
y12 2+ 782.84198
y8+ 972.47656
y14 2+-P 846.41583
y18 2+-P 1080.02649
b11+ 1179.61938
y18 2+ 1129.02441
973.46594
b18 2+, y9+-P 1055.51831
y2+ 246.15575
y4 2+-NH
b10 2+-H2
1315.56506
y12+-P 1466.68811
y14+-P 1691.81458

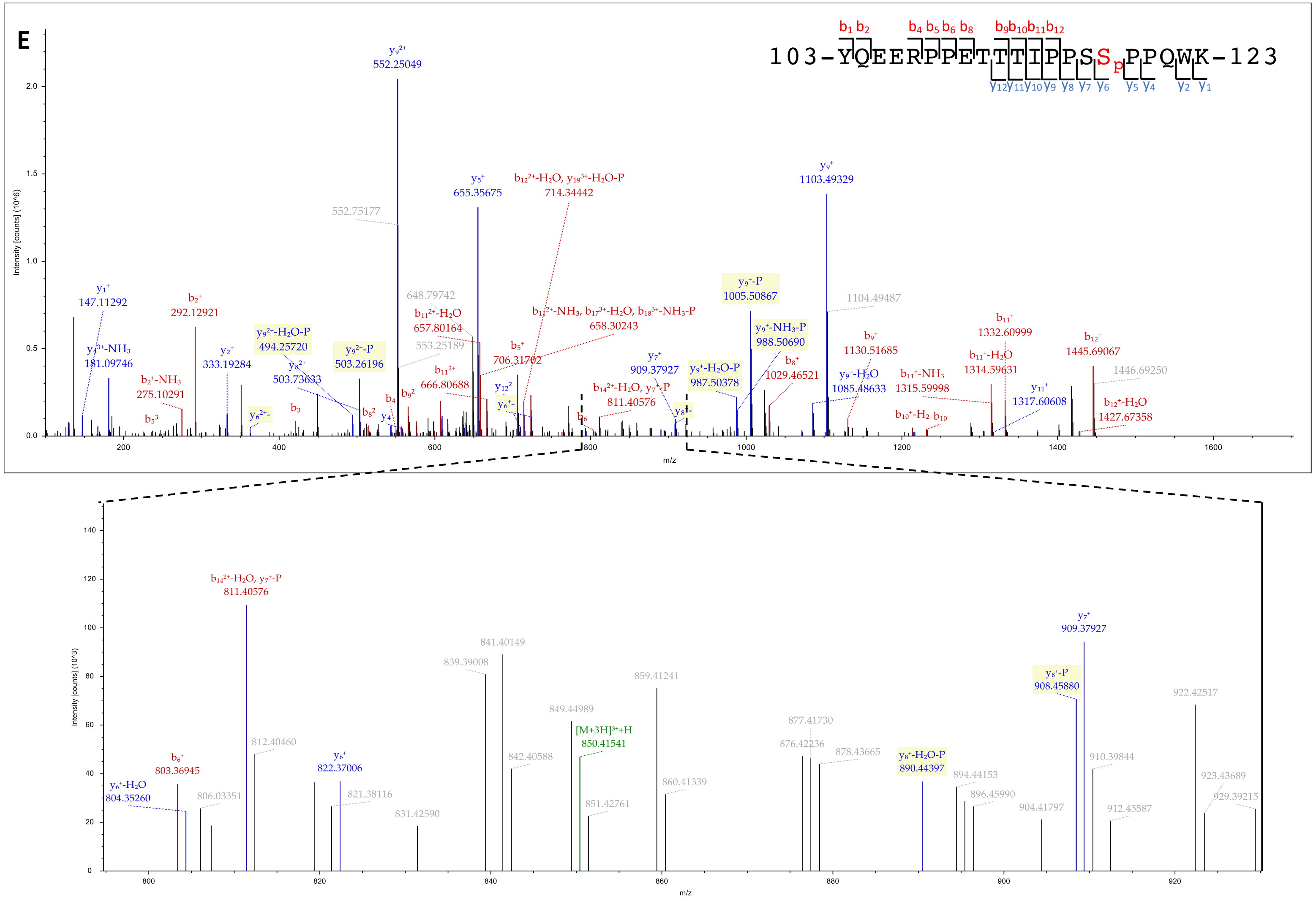

E
103-YQEERPPETTTIPPSSpPPQWK-123
Intensity [counts] (10^6)
m/z
Intensity [counts] (10^3)
m/z

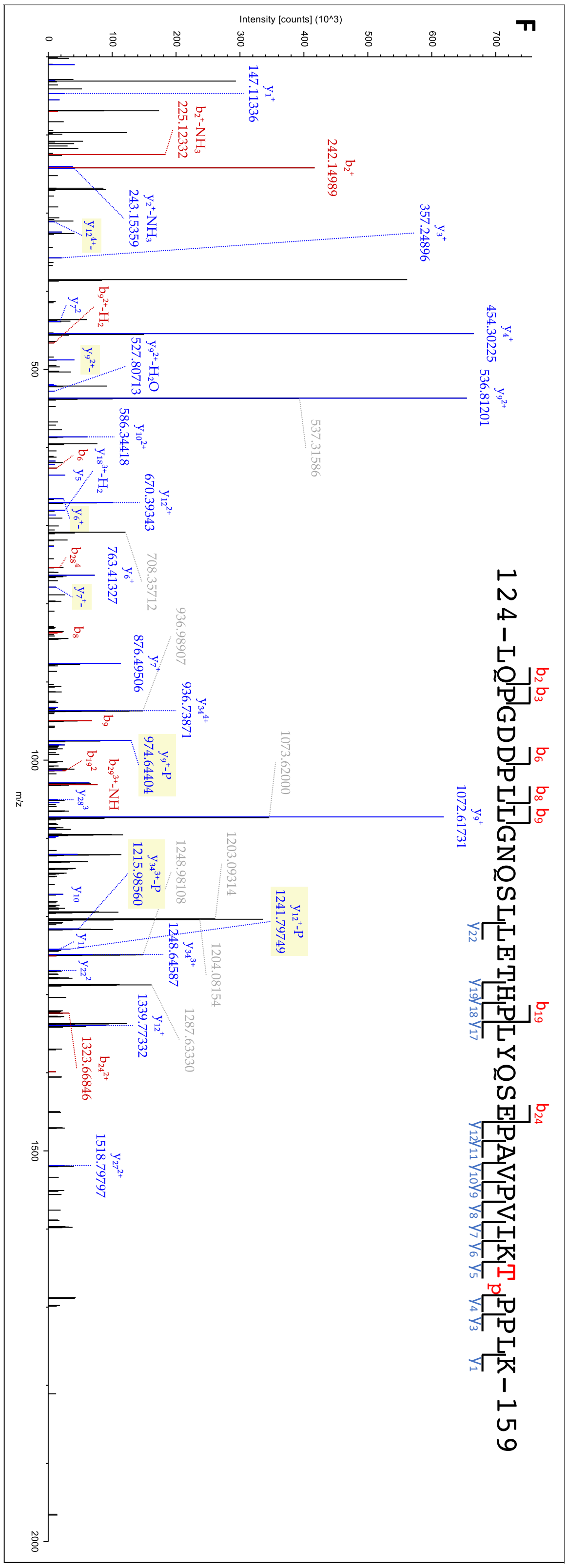

G

Phosphorylation position	Annotated Sequence	Modifications in Master Proteins	# PSMs	Positions in Master Proteins	# Missed Cleavages	Theo. MH+ [Da]	Confidence MS Amanda 2.0	Confidence Sequest HT	XCorr : Sequest HT	Amanda Score : MS Amanda 2.0
S8	[M].GQHPAKSpMDVR.[R]	P03145 1xPhospho [S8]	1	P03145 [2-12]	1	1321,5719	High	n/a	x	185,06
S76	[R].VGLSpNPTPQEIPQPQWTPEEDQKAR.[E]	P03145 1xPhospho [S]	8	P03145 [73-97]	1	2925,3778	High	High	4,9	497,56
T79	[R].RVGLSNPTpPQEIPQPQWTPEEDQK.[A]	P03145 1xPhospho [T79]	2	P03145 [72-95]	1	2854,3407	High	High	2,82	274,41
T89	[R].RVGLSNPTPQEIPQPQWTpPEEDQKAR.[E]	P03145 1xPhospho [T]	4	P03145 [72-97]	2	3081,479	High	High	3,48	405,54
S118	[R].YQEERPPETTTIPPSSpPPQWK.[L]	P03145 1xPhospho [S]	35	P03145 [103-123]	0	2548,1756	High	High	4,19	436,57
T155	[K].LQPGDDPLLGNQSLLETHPLYQSEPAVPVIKTpPPLK.[K]	P03145 1xPhospho [T155]	3	P03145 [124-159]	1	3985,0722	High	High	5,12	293,13

Fig. S2. Mass spectrometry analysis identifying phosphorylation sites of DpreS in DHBs L. Shown spectra correspond to the analysis of the upper band of the purification as shown in Fig. 1 and Fig 2a. The identified phosphorylation sites are indicated in red. A-F) Phosphorylation sites at residue S8 (A), S76 (B), T79 (C), T89 (D), S118 (E), and T155 (F) are displayed. G) Table showing, for each peptide: the phosphorylation position, the number of peptide spectrum matches (PSM) and missed cleavages, the theoretical peptide mass (Theo. MH+), the confidence and identification score using the MS Amanda 2.0 algorithm, and the confidence and Xcorr value of identification with the SEQUEST HT algorithm.
